# MiR-92b inhibitor promoted glioma cell apoptosis via targeting *DKK3* and blocking the Wnt/beta-catenin signaling pathway

**DOI:** 10.1186/1479-5876-11-302

**Published:** 2013-12-11

**Authors:** Qifeng Li, Ke Shen, Yang Zhao, Chenkai Ma, Jianwen Liu, Jie Ma

**Affiliations:** 1 Department of Pediatric Neurosurgery, Xinhua Hospital, Shanghai Jiaotong University, School of Medicine, Shanghai 200092, P R China; 2 State Key Laboratory of Bioreactor Engineering & Shanghai Key Laboratory of Chemical Biology, School of pharmacy, East China University of Science and Technology, #268, 130 Meilong Road, Shanghai 200237, P R China

**Keywords:** miR-92b, *DKK3*, The Wnt/beta-catenin signaling pathway, Glioma, Prognosis

## Abstract

**Background:**

MiR-92b was upregulated in gliomas. However, the association of miR-92b with glioma cell apoptosis and survival remains unknown.

**Methods:**

Proliferation capability of glioma cells upon tranfection with miR-92b mimics or inhibitors was detected by mutiple analyses, including MTT assays, colony formation assay. Apoptosis abilities of glioma cells were detected by flow cytometric analysis. The target of miR-92b was determined by luciferase reporter and western blot. The association of miR-92b with outcome was examined in twenty glioma patients.

**Results:**

MiR-92b expression was significantly increased in high-grade gliomas compared with low-grade gliomas, and positively correlated with the degree of glioma infiltration. Over-expression of miR-92b increased cell proliferation, whereas knockdown of miR-92b decreased cell proliferation via modulating the levels of the target, Target prediction analysis and a dual luciferase reporting assay confirmed that the inhibitory protein-coding Dickkopf-3 gene (*DKK3*) was a direct target of miR-92b. Furthermore, miR-92b could regulate the expression of downstream genes of the Wnt/beta-catenin signaling pathway, such as Bcl2, c-myc and p-c-Jun, in glioma cells. Finally, the increased level of miR-92b expression in high-grade gliomas confers poorer overall survival.

**Conclusions:**

The present data indicates that miR-92b directly regulate cell proliferation and apoptosis by targeting *DKK3* and act as prognostic factors for glioma patients.

## Introduction

A glioma is the most common form of neural malignancy. High grade glioma, especially glioblastoma, is a leading cause of brain cancer fatality involving highly invasive and neoplastic growth. Despite therapeutic advances, many patients suffer from tumor recurrence due to chemo- and radio- therapy resistance [1-3]. Increasing evidence suggests that the progression of a glioma is relative to the rate of both cell proliferation and apoptosis. Therefore, understanding the main regulatory mechanism of gliomas is key to the development of effective therapeutic approaches against this malignancy.

MicroRNAs (miRNAs) are small, endogenous, non-coding RNA molecules, which usually result in gene silencing by binding to complementary sequences in the three prime untranslated regions (3-UTRs) of target messenger RNA transcripts (mRNAs) [4-9]. The deregulation of miRNAs has been observed in various types of human malignancies, including lymphoma, colorectal cancer, lung cancer, breast cancer, papillary thyroid carcinoma, hepatocellular carcinoma and glioblastoma [10-13]. Accounting for approximately 1% of all the expressed human genes, miRNAs are predicted to regulate the expression of up to 1/3 of human protein-coding genes [14-17]. A few studies suggest that the downregulation of miRNAs may play a critical role in cancer progression by affecting not only proliferation but also apoptosis [18-20]. Primary brain tumors expressed higher levels of miR-92b than both primary tumors in other tissues and their metastases to the brain [21]. In neuroblastoma, mir-92b was reported to modulate the expression of the inhibitory protein-coding Dickkopf-3 gene (*DKK3*) [22]. However, the underlying mechanism of mir-92b in gliomas has not been identified so far.

In the current study, we demonstrate that high levels of miR-92b expression in gliomas confer highly aggressive invasion and poorer overall survival. Knockdown of miR-92b decreased glioma cell prolifirelation, reduced apoptosis and up-regulated the expression of the target, *DKK3*, whereas ectopic expression of miR-92b exhibited the opposite effects. Furthermore, miR-92b could regulate the expression of downstream genes of the Wnt/beta-catenin signaling pathway, such as Bcl2, c-myc and p-c-Jun. These findings indicate that *DKK3* is a critical target of miR-92b and that the microRNA could be critical therapeutic targets and survival predictors in glioma.

## Materials and methods

The human glioma tissue samples and their corresponding nontumorous tissues were collected at the time of surgical resection at the Department of Pediatric Neurosurgery, Xinhua Hospital, Shanghai Jiao Tong University. Twenty frozen glioma specimens with clinical data were collected from January 2008 to June 2013, including 9 grade I-II tumors, 8 grade III tumors and 3 grade IV tumors. The glioma samples were deep-frozen using liquid nitrogen, stored at −80°C and were quantified by Real-time PCR. This study was approved by the Institutional Review Board of Xinhua hospital. Patients were followed by clinical and laboratory monitoring on a regular basis starting at definitive diagnosis. Disease-specific survival time was defined as the time from definitive diagnosis to disease-specific death.

### Reagents

The antibodies aganist c-jun, phospho-c-jun, JNK, phospho-JNK, *DKK3*, beta-catenin, Bcl-2, β-actin, caspase-3, Bax, c-myc were purchased from Santa Cruz Biotechnology (California, USA). The dual luciferase reporter assay system, the PGL3-Promoter, the PGL3-Basic and PRL-TK vectors were purchased from Promega (Promega Corporation, Wisconsin, USA). The miRNA mimics and siRNA were purchased from Biomics Biotechnologies (Nantong, China). All other chemicals were from Sigma- Aldrich unless otherwise stated.

### Cell cultures and transfection

The human glioma cell lines U251, U87, A172 and SHG44, and human astrocytes (HA) (Cell Bank of the Chinese Academy of Science, Shanghai, China), were maintained in RPMI 1640 medium (Gibco Industries, Inc. Carlsbad, CA) with 10% fetal bovine serum (Gibco Industries, Inc.) at 37°C in a humid atmosphere wih 5% CO2. Cell transfection was performed using Lipofectamine 2000 (Invitrogen) according to the manufacturer’s instructions.

### MicroRNA microarrays

Total RNA was extracted from eight glioma tissues using the miRVana miRNA Isolation Kit (Ambion, Carlsbad, USA) according to the manufacturer’s instructions. The samples were subsequently submitted to Shanghai Biotechnology Corporation (Shanghai, China) for array hybridization on an Agilent Human miRNA array (v.12.0). Each microarray chip was hybridized with a single sample labeled with either Cy3 or Cy5. Background subtraction and normalization were performed. The raw data were deposited at Shanghai Biotechnology Corporation (Shanghai, China) and have not been reported publicly up till the present moment. We selected the miRNAs that exhibited a difference in expression levels of at least 2-fold (p<0.05) between the glioma tissue samples and their corresponding nontumorous tissues.

### RNA extraction and quantification

Total RNA was extracted using Trizol (Invitrogen Corporation, California, USA) according to the manufacturer’s instructions. Reverse transcription was performed using One Step PrimeScript®miRNA cDNA Synthesis Kit (Takara Bio Inc, Dalian, China). Real-time PCR was performed using SYBR®Premix Ex TaqTM II (Takara Bio Inc, Dalian, China) with an iCycler®thermal cycler (Bio-Rad, Hercules, USA).U6 RNA was used as a miRNA internal control. The primers of miR-92b was as follows: 5′-TATTGCACTCGTCCCGGCCT-3′.

### Colony formation assay

U251 and U87 cells were transfected with miR-92b mimics, a control oligonucleotide and a miR-92b inhibitor. After the transfection, the U251 and U87 cells were counted and seeded in 12-well plates (in triplicates) at a density of 50 and 60 cells per well, respectively. The culture medium was replaced every 3 days. The number of colonies was counted on the sixth day after seeding. The rate of colony formation was calculated with the following equation: colony formation rate (%) = (number of colonies number of seeded cells) × 100%.

### MTT assays

The MTT assay was used to determine cell viability. All the cells were seeded into 96-well culture plates (2×103 cells/well) in regular growth medium. The cells transfected with miR-92b mimics. control and inhibitors were grown for 4 days. One plate was developed immediately after the medium change and other plates were developed every 24 hours for 4 days. Assays were initiated by adding 20 L of MTT substrate to each well and incubating the cells for an additional 3 hours. Finally, the medium was removed and 200 L DMSO was added to each well. The absorbance was measured at 492 nm using an Automated Microplate Reader (Multiskan Ex, Lab systems, FIN).

### RT-PCR

Analysis was used to determine the relative expression levels of miRNAs. Total RNA was isolated using TRIZOLTM reagent (Promega Corporation, Wisconsin, USA) according to the supplier’s instruction. Reverse transcription was done using One Step PrimeScript® miRNA cDNA Synthesis Kit (TaKaRa Biotechnology Ltd, Shandong, CHN). Real-time PCR was performed using SYBR Green Supermix with an iCycler®thermal cycler (Bio-Rad Laboratories Inc, California, USA).Primers of all genes were in Supple-mentary. The data were collected and analyzed using the comparative Ct (threshold cycle) method using GADPH as the reference gene.

### MicroRNA target prediction

The target genes of miR-92b were predicted by the following computer-aided algorithms: TargetScan Human Release 6.2 (http://www.targetscan.org).

### Luciferase assay

The 3′UTR of human *DKK3*, containing the putative target sites for miR-92b, was amplified by PCR. The wild-type and mutant inserts were transfected into the PGL3-promoter vector. Dual-Luciferase reporter assays were performed according to the manufacturer’s instructions (Promega, Madison, WI) as previously described [23].

### Flow cytometric analysis of apoptosis

Cells (6 *104/well) were plated in 6-well plates in antibiotic-free medium and transfected with control oligonucleotide (100 nM) or inhibitor (100 nM) using Lipofectamine 2000 (Invitrogen 4 Corporation, California, USA) according to the manufacturer’s recommendation. Luciferase and renilla signals were measured 48 h after transfection using the Annexin V-FITC apoptosis detection kit (Invitrogen Corporation, California, USA) as described by the manufacturer’s instructions. Three independent experiments were performed and the data are presented as the mean + SD.

### Western blot analysis

Total cell lysates (50 ug) were fractionated by SDS/PAGE. The proteins were electroblotted onto nitrocellulose membranes and Western blot analyses were carried out according to standard procedures as previously described [24]. β-actin was used as the loading control in the Western blots.

### Statistical analysis

The statistical analyses were performed using the Statistical Package for the Social Sciences software using the two-tailed Student’s t-test. The significance was determined at the 95% confidence interval. All the data were expressed as the mean ± standard deviation (SD) from a representative experiment.

## Results

### Expression and clinical significance of miR-92b in gliomas

**Figure 1:**
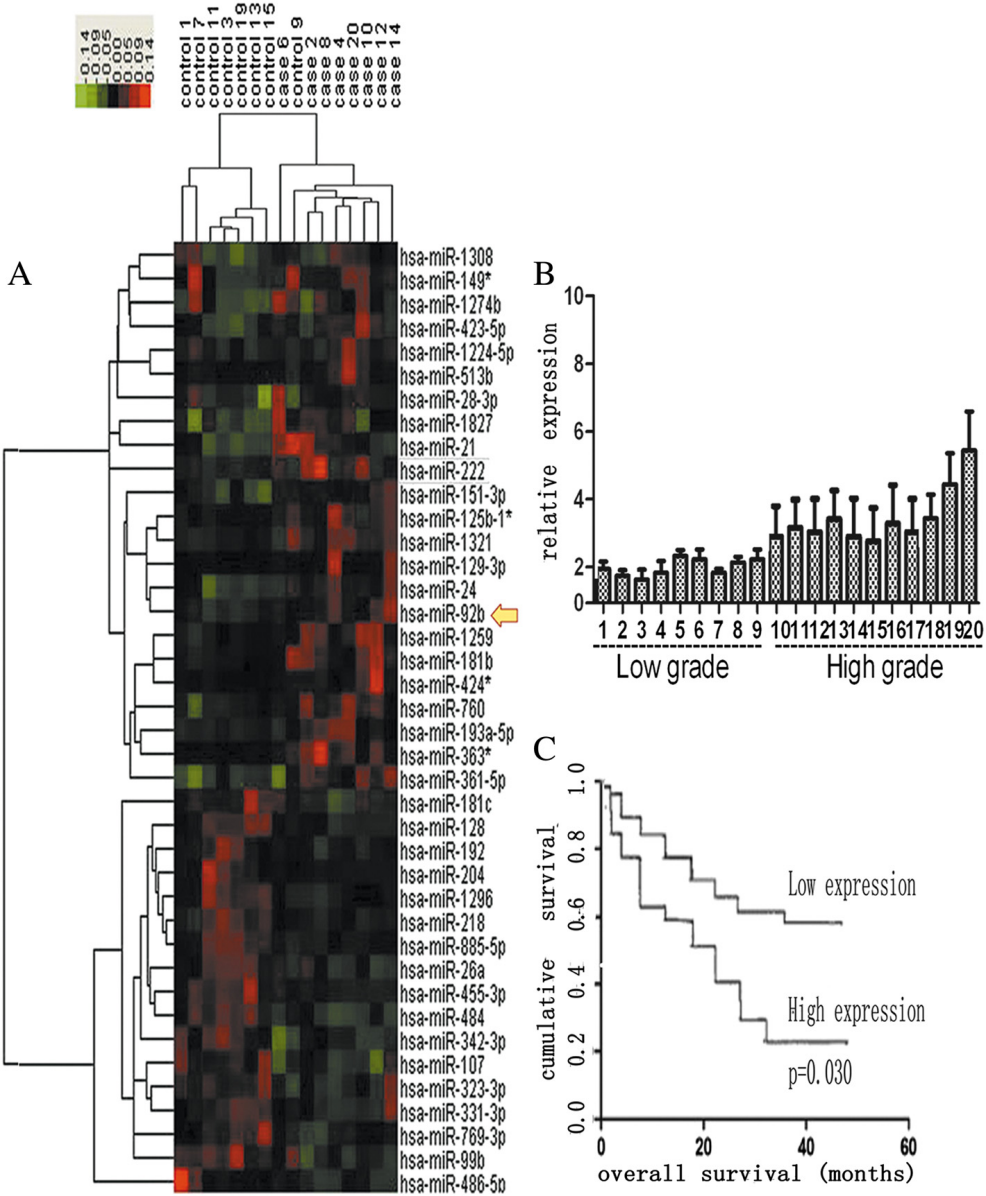
**Expression and clinical significance of miR-92b in gliomas. (A)** A heat map diagram generated by unsupervised clustering analysis with 40 significantly deregulated miRNAs in eight human glioma tissues. The hierarchical clustering was performed with the average linkage and uncentered correlation. The miRNA expression profile effectively segregated the human glioma samples from their corresponding nontumorous tissues (frozen samples) (p<0.01). The red and green colors represent the high and low expression, respectively. **(B)** miR-92b expression in 20 glioma tissues assayed by real-time PCR. **(C)** Kaplan-Meier survival curves for miR-92b expression. High-grade glioma patients with high levels of miR-92b had a significantly worse outcome. The expression level was categorized as low expression (final score 3) and high expression (>3). The number of patients in each group was shown: miR-92b, low expression (9 patients) and high expression (11 patients).

To identify the miRNAs that are potentially involved in gliomas, we first examined the miRNA expression profiles in 8 glioma tissues and their corresponding nontumorous tissues using Agilent Human miRNA array (v.12.0), which consists of 873 capture probes for mature human miRNAs. After the microRNA expression was normalized by U6 expression, The microarray results showed that 20 miRNAs were significantly overexpressed (fold change > 2, p<0.05) in the glioma tissues (T) compared with their corresponding normal tissues (N). On the other hand, 20 miRNAs were underexpressed significantly (fold change > 2, p<0.05) (Figure 1A), The data have not been reported publicly up till the present moment. Because the downregulated miRNAs have been studied by our colleagues, we chose the upregulated miRNAs for further study. At present, gliomas are classified as four grades from grade I to grade IV. Gliomas with grade I and grade II are classified as low grade gliomas, whereas gliomas with grade III and IV are classified as high grade gliomas. Generally, comparing with high grade gliomas, low grade gliomas have good results, because low grade gliomas have less invasiveness. In our experiments, we performed real-time PCR for quantitative analysis of miR-92b in 20 glioma tissues. MiR-92b expression was significantly increased in high-grade gliomas compared with low-grade gliomas, and a similar trend for miR-92b was detected (Figure 1B). We also analyzed the overall survival of 20 patients. The Kaplan-Meier curves for patient according to miR-92b expression levels in the glioma tissues are shown in Figure 1C. The increased expression of miR-92b was significantly associated with a poor overall survival (p=0.030).

### A miR-92b Inhibitor Impeded Cell Viability and Colony Formation and Promoted Apoptosis

**Figure 2:**
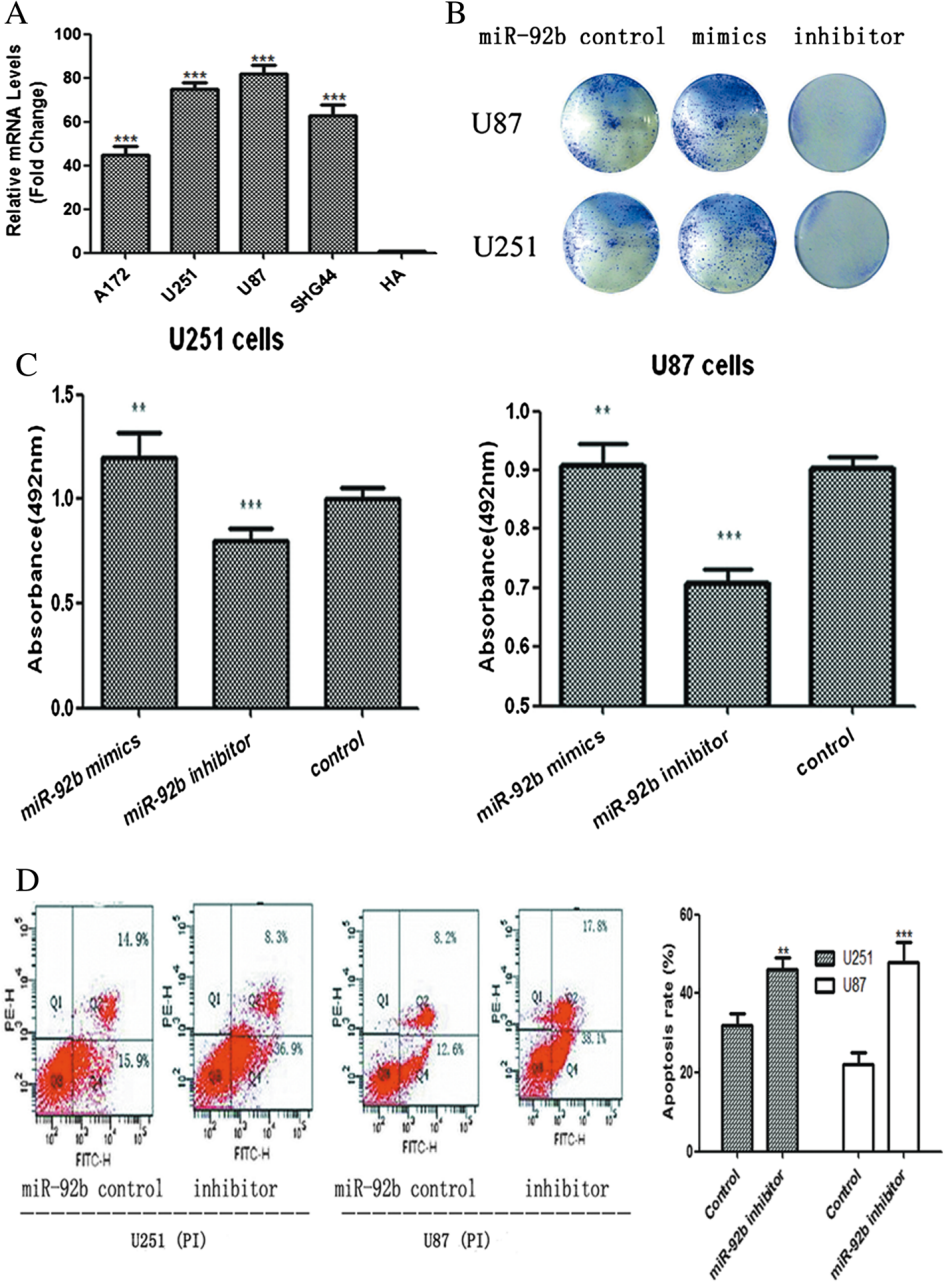
**The effects of miR-92b on proliferation and apoptosis in U251 and U87 cells. (A)** Realtime PCR analysis of miR-92b expression in human astrocyte cell line (HA) and glioma cell lines (including U251, U87, SHG44 and A172). The average miR-92b expression was normalized by U6 expression. Each bar represents the mean of three independent experiments. ***p<0.001. **(B)** After transfection, U251 and U87 cells were counted and seeded separately in 12-well plates. The colonies were counted on the sixth day after seeding and the rate of colony formation was calculated. All the data were representative of three independent experiments. **(C)** The rates of cell growth were measured using an MTT assay. All the cells were seeded into 96-well culture plates. The absorbance at 492 nm was measured. MiR-92b mimics increased cell viability (**p<0.01). Conversely, miR-92b inhibitors showed a significant decrease in cell viability (***p<0.001). All the data were presented as the mean ± SD of a representative average of three independent experiments. **(D)** Three days after the transfection, the apoptosis was assessed using Annexin V-FITC analysis. Compared to the control oligonucleotide group, a significant increase in cellular apoptosis was inducted by miR-92b inhibitors, whereas the miR-92b mimics group showed a decrease in cellular apoptosis. The bar chart represents our repeating results. All data were presented as means ± SD and as representative of an average of three measurements.

To confirm miR-92b overexpression in glioma, we quantitated the expression of miR-92b in four glioma cell lines, U251, U87, SHG44 and A172, and in a human astrocyte cell line (HA). The results showed that miR-92b expression was significantly higher in the glioma cells than in the human astrocyte cell line (HA) (Figure 2A). And the U251 and U87 glioma cell lines, which possessed the highest levels of miR-92b expression among all tested glioma cell lines, were selected for further studies. Because the miR-92b expression was higher in the gliomas than in the corresponding nontumorous tissues, we hypothesized that the downregulation of miR-92b could promote apoptosis and impede proliferation. Two glioma cell lines, U251 and U87, were transfected with either miR-92b mimics, a control oligonucleotide or a miR-92b inhibitor (antisense oligonucleotide-miR-92b) to assess the effect of miR-92b in glioma cells. The miR-92b inhibitor impeded colony formation, compared to the miR-92b mimics (Figure 2B). Then, we performed an MTT assay and found that the miR-92b inhibitor could reduce the viability of the glioma cells significantly (p<0.001), whereas the miR-92b mimics could promote their viability (*p*<0.05) (Figure 2C). Because the miR-92b inhibitor could impede cell viability, we were interested in finding out whether it could promote apoptosis. We used the Annexin V-FITC analysis to assess the rate of apoptosis. In the U251 cells, the miR-92b inhibitor caused apoptosis (45.2%), compared to the control group (30.8%). In the U87 cells, the apoptosis rate was 55.9% with the miR-92b inhibitor, compared to the control group (20.8%) (Figure 2D). The bar chart represents our repeating results. All data were presented as means ± SD and as representative of an average of three measurements.

### MiR -92b directly targeted the *DKK3* 3′UTR

**Figure 3:**
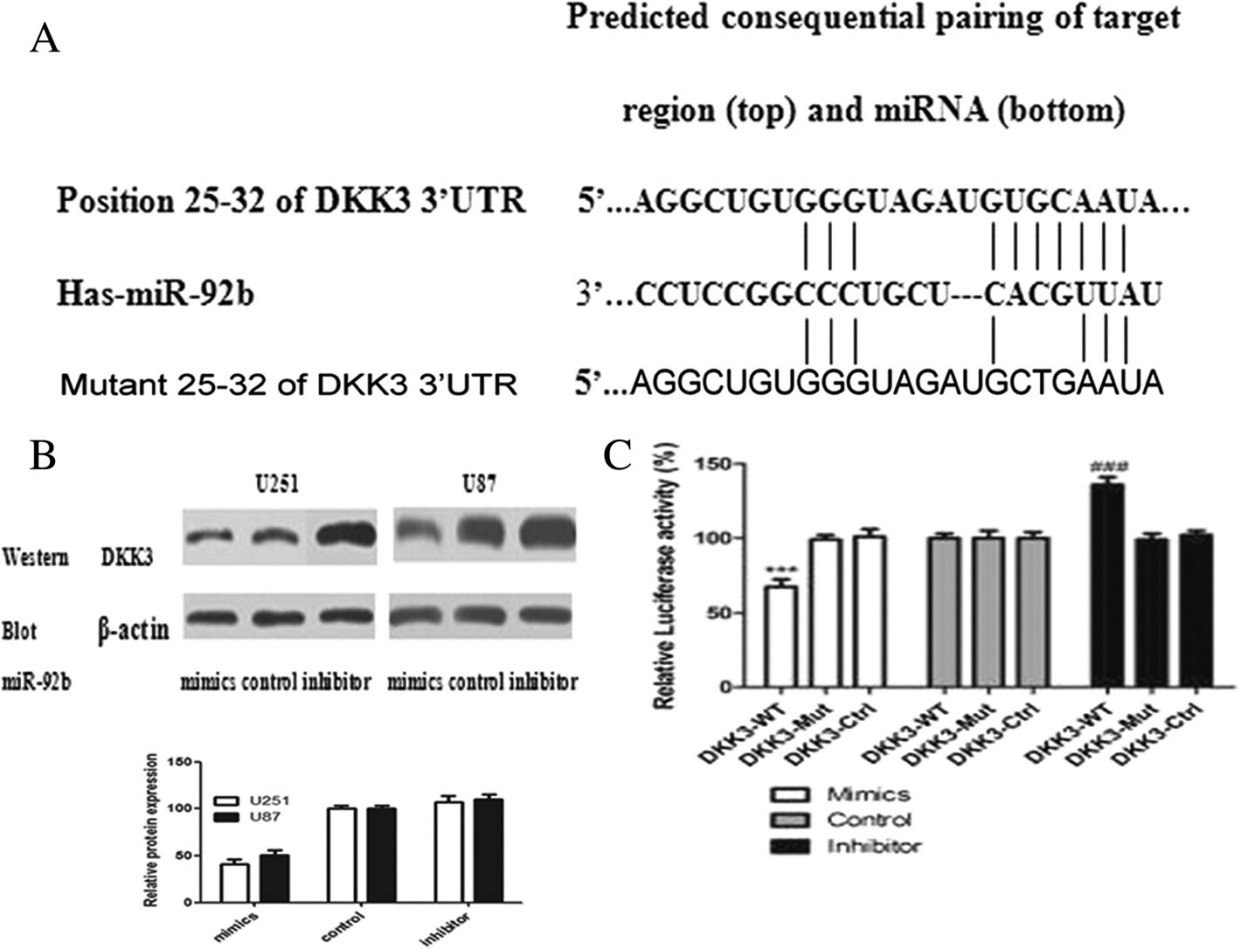
***DKK3 *is a direct target of miR-92b. (A)** The putative miR-92b-targeted sequence in the *DKK3* gene. TargetScan predicts the binding site to be in the 3′-UTR of *DKK3*. **(B)** The *DKK3* protein level was assessed 48 h after transfection of U251 and U87 cells with either the miR-92b mimics (100 nM), the control oligonucleotide (100 nM) or the miR-92b inhibitor (100 nM). *DKK3* protein levels were detected by western blot assays. β-actin protein was assayed as a control. Scanning densitometry of the blots was used to quantify the Western blotting data. (n=3; means ± SEM **p<0.01, ***p<0.001 t-test). All the data were presented as a representative average of three independent experiments. **(C)** The U87 cells transfected with the vector containing the *DKK3* 3*'*-UTR fragment with the miR-92b binding sequence had inhibited luciferase activity after miR-92b transfection. No luciferase activity change was observed when the cells were transfected with the plasmid containing a *DKK3* 3′-UTR fragment without the miR-92b binding sites. Each bar represents the mean of three independent experiments. ***p<0.001.

To assess how the miR-92b inhibitor contributed to the apoptosis in glioma cells, we investigated the potential gene targets of miR-92b with the help of the prediction tool TargetScanHuman Release 6.2. Hundreds of different targets were predicted and the genes involved in migration, invasion or apoptosis were selected as the potential relevant targets of miR-92b. One of these genes, *DKK3* (Figure 3A), is regarded as a secreted antagonist of the Wnt/beta-catenin signaling pathway [25,26]. Because this pathway is always activated in gliomas [27-29], we hypothesized that the miR-92b inhibitor could play a pro-apoptotic role by inhibiting the Wnt/beta-catenin signaling pathway.

To test our hypothesis, we analyzed the protein levels of *DKK3* and miR-92b in the glioma cells. The results showed a negative correlation between the levels of miR-92b and *DKK3* in the glioma cells (Figure 3B). We then decided to test whether *DKK3* is a direct target of miR-92b. We first constructed a luciferase reporter in which the nucleotides of the *DKK3*-3′UTR complementary to miR-92b were inserted into the 3′UTR of PGL3-promoter vector (*DKK3*-WT). Correspondingly, we also generated both a mutant reporter (*DKK3*-Mut), in which the sequence in the miR-92b seed region complementary sites was changed (the sequence of the mutated construct is 5′…AGGCUGUGGGUAGAUGCTGAAUA…), and a control reporter (*DKK3*-Ctrl), which contained a non-related fragment of cDNA. MiR-92b over-expression plasmid was co-transfected with *DKK3*-WT or *DKK3*-Mut or *DKK3*-Ctrl into cells. The assays showed that the luciferase activity in the *DKK3*-WT transfected cells significantly decreased compared to the luciferase activity in the mutant and negative control cells and vice verse, suggesting that miR-92b reduced the luciferase activity of *DKK3*-WT but had no effect on *DKK3*-Mut (Figure 3C). Therefore, we concluded that the *DKK3* is the target of miR-92b.

### MiR-92b inhibitor impeded the Wnt/beta-catenin signaling pathway by targeting *DKK3*

**Figure 4:**
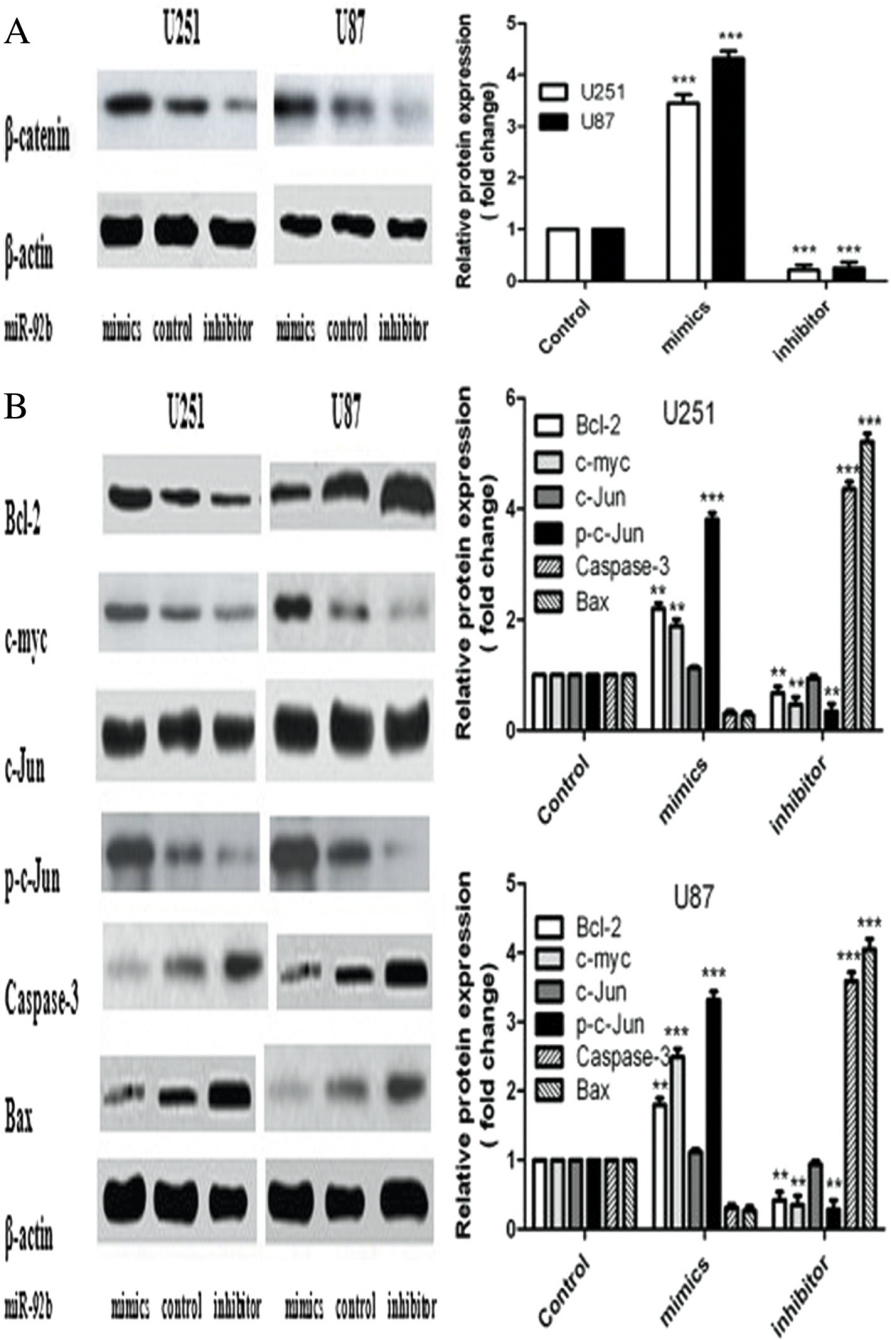
**The alternation of the Wnt/beta-catenin signal pathway.** The U251 and U87 cells were transfected with either the miR-92b mimics (100 nM), the control oligonucleotide (100 nM) or the miR-92b inhibitor (100 nM). **(A)** The beta-catenin protein levels were assessed 48 h after the transfection. β-actin was used as the loading control. The Western blots are representative of three independent experiments. Scanning densitometry analysis of the blots was used to quantify the data. **(B)** Bcl2, c-myc, c-Jun (total and phosphorylated), Caspase-3 and Bax protein levels were assessed 48 h after transfection with either the miR-92b mimics, the control oligonucleotide and the miR-92b inhibitor. β-actin was used as the loading control. Scanning densitometry analysis of the blots was used to quantify the data. The data were expressed as the mean ± SD and were representative of an average of three independent experiments. Significant differences from the control were indicated by **p <0.01 and ***p <0.001.

Because *DKK3* is a critical antagonist of the Wnt/beta-catenin signaling pathway, and miR-92b could inhibit the expression of *DKK3*, we hypothesized that miR-92b could modulate the Wnt/beta-catenin signaling pathway via beta-catenin. To determine this, beta-catenin protein levels were evaluated by Western blotting in cells treated with either the miR-92b mimics, the control oligonucleotide or the miR-92b inhibitor. The data showed that the miR-92b mimics significantly promoted the expression of beta-catenin, whereas the miR-92b inhibitor inhibited the expression of beta-catenin (*p*<0.001) (Figure 4A). Furthermore, we tested the protein levels of the downstream genes Bcl2, c-myc, c-Jun, phospho-c-Jun and the pro-apoptotic genes Caspase-3 and Bax by Western blotting. The results showed that the miR-92b inhibitor could modulate the expression of these genes and that it reduced the expression of Bcl2 greatly. Bcl-2 is not only a downstream gene of the Wnt/beta-catenin signaling pathway but also an anti-apoptotic gene. To test how miR-92b stimulated apoptosis, we also analyzed the apoptotic genes including Caspase-3 and Bax. The results showed that Caspase-3 was activated in the cells treated with the miR-92b inhibitor (Figure 4B).

## Discussion

MicroRNAs play a crucial role in the process of tumor formation. They impact the dynamic balance between oncogenes and tumor suppressor genes by degrading target genes, thereby contributing to cancer progression [30]. Previous studies have shown that miR-92b is over-expressed in brain primary tumor, as compared to primary tumors from other tissues and their metastases to the brain [21]. Based on topological and functional analyses, it was also reported that miR-92b could play important roles related to the Notch signaling pathway in Glioblastoma multiforme (GBM) tumors [31]. However, there were no reports about the association of miR-92b and survival.

In our study, we focused on the regulatory mechanisms of the miR-92b in gliomas. Initially, the miRNA array results showed that miR-92b was upregulated in gliomas, which suggested that miR-92b could play an important role in the development of gliomas as an oncogene. Thus, we hypothesized that the downregulation of miR-92b could promote apoptosis, providing a potential strategy for glioma treatment. In vitro, our studies demonstrated that the miR-92b inhibitor significantly promoted apoptosis and impeded cell viability and colony formation. To determine how miR-92b was involved in the development of gliomas, we used TargetScan and predicted that *DKK3* was a probable target of miR-92b in the 3!UTR of *DKK3*. We proved that the miR-92b overexpression resulted in the downregulation of *DKK3* at the protein level, whereas the functional inhibition of miR-92b led to the inhibition of *DKK3*, strongly suggesting that *DKK3* is regulated by miR-92b in gliomas. Meanwhile, a dual luciferase reporter assay identified *DKK3* as a direct target of miR-92b.

*DKK3* is a critical antagonist of the Wnt/beta-catenin signaling pathway [32], which has been shown to be inhibited by miR-92b in neuroblastomas, but the mechanism in gliomas has not been elucidated fully [22]. A previous study showed that the Wnt/beta-catenin signaling pathway was activated in gliomas [33]. Thus, we speculated that miR-92b played its role by regulating the Wnt/beta-catenin signaling pathway. To elucidate the mechanism, we detected the protein level of beta-catenin and the downstream genes of the Wnt/beta-catenin signaling pathway, such as Bcl2, c-myc, c-Jun and p-c-Jun. The results showed that the overexpression of miR-92b inhibited *DKK3* and increased the expression of beta-catenin (Figure 4A), which suggested that miR-92b modulated beta-catenin via *DKK3*. To verify whether miR-92b could modulate the Wnt/beta-catenin signaling pathway, we measured the expression of the downstream genes Bcl2, c-myc, c-Jun and p-c-Jun by Western blotting. The results showed that the miR-92b inhibitor could modulate the expression of these genes. The protein expression of Bcl-2, which is not only a downstream gene of the Wnt/beta-catenin signaling pathway but is also an anti-apoptotic gene, was inhibited by miR-92b. This demonstrated that miR-92b could modulate the genes downstream of the Wnt/beta-catenin signaling pathway. Furthermore, it could modulate apoptosis. To testify how miR-92b affected apoptosis, we analyzed the apoptotic genes Caspase-3 and Bax. The results demonstrated that miR-92b increased the expression of Caspase-3 and Bax, indicating that Caspase-3 was activated after treatment with the miR-92b inhibitor (Figure 4B).

Recent data showed miR-92b could regulate Wnt/beta-catenin signaling via Nemo-like kinase [34]. However, the significance of miR-92b in prognostic determination have not been shown in glioma. In this study, our data suggest that a high miR-92b expression level might be a valuable marker for pathological diagnosis and prognosis prediction in high-grade glioma; high miR-92b expression levels were significantly associated with poor survival in high-grade glioma patients as determined by Kaplan-Meier analysis.

In summary, our data demonstrated that the miR-92b could regulated glioma cell proliferation, apoptosis by directly targeting *DKK3*. We also provide direct evidence that high levels of miR-92b expression are significantly associated with poorer overall survival. To conclude, our data suggest that miR-92b could be a intrinsic regulator of progression in glioma cells and could function as a potential target and predictor of survival in glioma.

## Competing interests

The authors declare that they have no competing interests.

## Authors’ contributions

QL identified and recruited patients and organised sample collection, performed quantitative RT–PCR. KS performed western bolt, cell proliferation and Luciferase assays. YZ and CM performed MTT assay and construct of vectors. JL and JM participated in the project design, coordination the experiments, and manuscript preparation. All authors read and approved the final manuscript.
